# Effect of heavy haze and aerosol pollution on rice and wheat productions in China

**DOI:** 10.1038/srep29612

**Published:** 2016-07-08

**Authors:** Xuexi Tie, Ru-Jin Huang, Wenting Dai, Junji Cao, Xin Long, Xiaoli Su, Shuyu Zhao, Qiyuan Wang, Guohui Li

**Affiliations:** 1 Key Laboratory of Aerosol Chemistry & Physics, SKLLQG, Institute of Earth Environment, Chinese Academy of Sciences, Xi’an, 710061, China; 2 Center for Excellence in Urban Atmospheric Environment, Institute of Urban Environment, Chinese Academy of Sciences, Xiamen, 361021, China; 3 Centre for Atmospheric and Marine Sciences, Xiamen Huaxia University, Xiamen 361024, China; 4 Laboratory of Atmospheric Chemistry, Paul Scherrer Institute (PSI), 5232 Villigen, Switzerland; 5 Institute of Global Environmental Change, Xi’an Jiaotong University, Xi’an, 710049, China

## Abstract

In China, regional haze pollution is a serious environmental problem. The impact on ecosystem, however, is not clearly understood. This study investigates the effect of regional haze pollution on the yields of rice and wheat in China. The spatial and temporal distributions of aerosol optical depth (AOD) show high particulate pollution in the North China Plain region, Yangtze River Delta region, the central eastern China, and the Si Chuan Basin, coexisted largely with crop growth in time and space. The solar irradiance reaching these regions is estimated to reduce by up to 28–49%, calculated using the AOD distributions and tropospheric ultraviolet-visible (TUV) model. Reduction of solar irradiance in these regions can depress optimal yields of about 45% of rice and 75% of wheat growth in China, leading to 2% reduction in total rice production and 8% reduction in total wheat production in China. However, there are large uncertainties of the estimate related to the diffuse solar radiation. For high diffuse radiation case, the estimate reductions of rice and wheat decrease to 1% and 4.5%, respectively. A further detailed study is needed to clearly understand this effect to meet the growing food demand in the nation in the coming decades.

Over the last 30 years, rapid urbanization and economic development in China have led to an increase in air pollution^1^. In particular, particulate pollution is a serious environmental problem, with PM_2.5_ (particulate matter with an aerodynamic diameter less than 2.5 μm) concentrations of 1–2 orders of magnitude higher than those observed in urban areas in the US and European countries^2^. For example, measurements at Beijing show that the hourly averaged aerosol concentrations often exceed 300 μg m^−3^, causing heavy haze events in recent winters^3^,^4^,^5^,^6^. The heavy aerosol pollutions have led to many environmental problems in China, such as human health, visibility and regional climate, which have been extensively studied^1^,^3^,^7^,^8^. However, the effects on ecosystem, such as the crop production, have not been systematically studied due to lack of measurements of horizontal and temporal distribution of aerosol in China. In this study, we first investigated the spatial and temporal distribution of aerosol in China using the satellite measurement results of the Moderate Resolution Imagine Spectroradiometer (MODIS) aboard Aqua satellite. A state-of-the-art Tropospheric Ultraviolet-Visible (TUV) model was then applied to calculate the reduction of the solar radiation due to the scattering and absorbing of aerosol particles. Filed experimental measurements of the relationship between the solar radiation and crop yields^9^,^10^,^11^,^12^,^13^,^14^ were used to estimate the effect of aerosols on the reductions of rice and wheat net yields in China. However, there are large uncertainties for estimating the effect of solar radiation on crop production, which related to the diffuse solar radiation^15^. Canopy photosynthesis tends to be significantly more light-use efficient under diffuse solar radiation than under direct solar radiation^16^. The uncertainties of diffuse solar radiation on the net yield of crop are studied under different diffuse radiation cases.

## Results and Discussion

**Figure 1:**
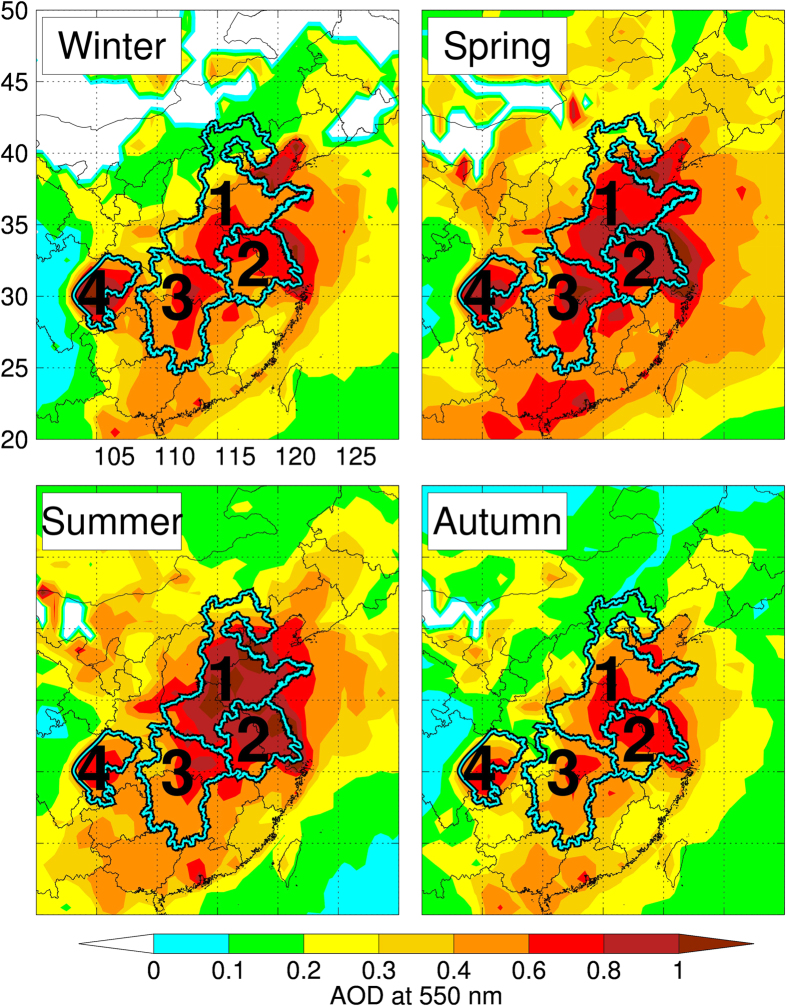
The MODIS AOD horizontal distributions in different seasons (Winter-Dec. Jan. Feb., Spring-Mar. Apr. May., Summer-Jun. Jul. Aug., and Autumn-Sep. Oct. Nov.). The outlines with numbers represent for different heavy aerosol pollution regions in China. 1 represents for the North China Plain region (NCP); 2 for the Yangtze River Delta region (YRD); 3 for the central eastern China (CEC); and 4 for the Si Chuan Basin (SCB). The map was generated by the IDL software version IDL 8.1 (Exelis, USA), http://www.exelisvis.com/.

Figure 1 shows the horizontal distributions of aerosol optical depth (AOD) which is a measure of the extinction of solar radiation by aerosol. High AOD values were observed mainly in four regions of China including the North China Plain region (NCP), the Yangtze River Delta region (YRD), the central eastern China (CEC), and the Si Chuan Basin (SCB). These AOD values (0.4–0.8) are significantly higher than those (~0.1) observed in the US and European countries^17^,^18^, suggesting severe aerosol pollution in these four regions. This is consistent with ground-based observations which show that the 24-hr integrated PM_2.5_ concentrations in China often exceed 100 μg m^−3^, ~3 times that of the US EPA standard. Such high aerosol concentration can cause significant reduction of solar radiation to the surface, which in turn may affect the photosynthesis of plants.

It is worth noting that the NCP, YRD, CEC, and SCB regions are also important crop production areas in China, as shown in Figure S1 (Supplementary Information). The detailed information of the wheat and rice production is listed in Tables S1 and S2 (Supplementary Information). The NCP region, including three important wheat production provinces (Hebei, Henan, and Shandong), shares 47% of the total wheat production in China. The YRD region includes two important rice and wheat production provinces (Anhui and Jiangsu), producing 15% of the total rice and 19% of the total wheat in China. The CEC region includes two important rice production provinces (Hunan and Hubei) with 20% of the total rice production in China, while the SCB region provides 7% of the total rice production in China. The co-location between the areas of high crop productions and of high aerosol concentrations rises an important question that how the aerosol pollution affects the solar radiation and therefore the crop production in China.

**Figure 2:**
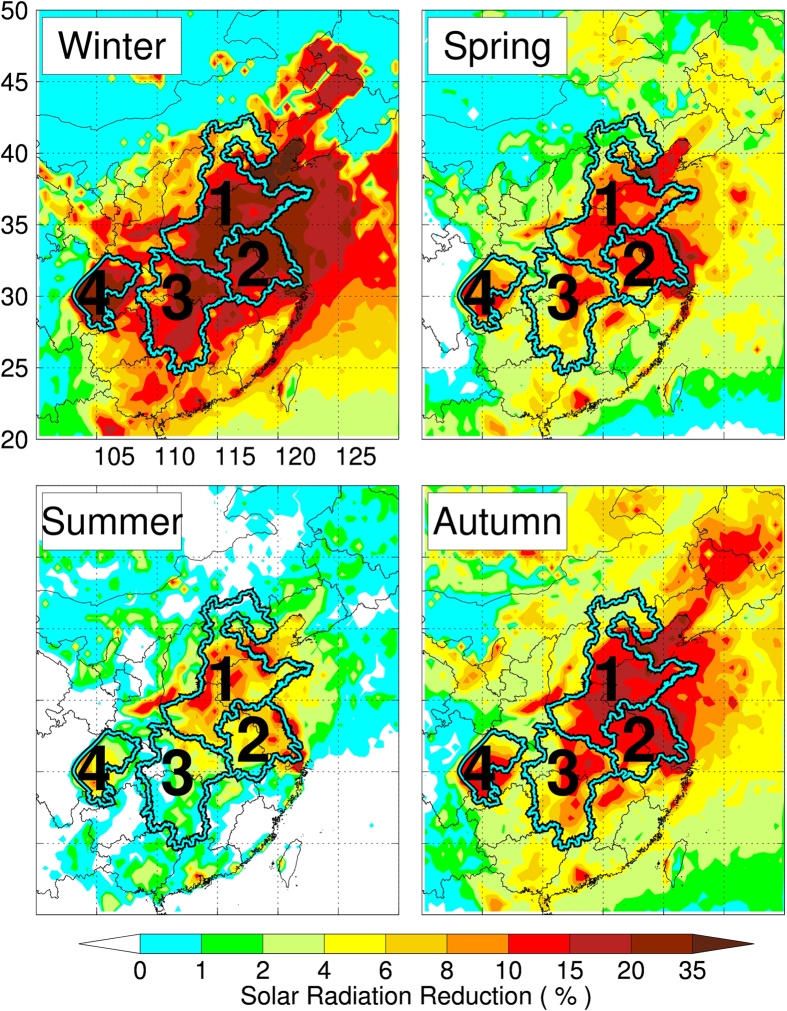
The calculated solar radiance reduction (%) at the surface, using the measured AOD distributions and the TUV model in different seasons. The reduction ranges between the 25^th^ and 75^th^ percentiles for all four regions are 11–29% in winter, 4–12% in spring, 1–9% in summer, and 5–16% in autumn. More details about the statistical data are shown in Supplementary Information Table S3). The map was generated by the IDL software version IDL 8.1 (Exelis, USA), http://www.exelisvis.com/.

The photosynthesis of crop plants is mainly dependent on three important factors, including water, nutrients, and sunlight^19^. In managed ecosystems such as those in cultivation for food crops, on the other hand, conditions are often manipulated to maximize crop yields through irrigation and fertilization. Thus the possibility that surface solar radiance can affect net yields of crops is far greater than other two factors. In order to calculate the effect of aerosols on solar radiative forcing, a state-of-the-art radiation transfer model (TUV) is applied. The TUV model is developed at the National Center for Atmospheric Research^20^,^21^, and has been widely used by the scientific community. Figure 2 shows the calculated reduction of solar radiation at the surface. The reduction in the four regions aforementioned is higher in winter (interquartile range [Q1–Q3] 11–29%) mainly due to the enhanced emissions of aerosol from domestic heating. The reductions of solar radiation in other seasons are also significant (interquartile range 5–16% in autumn, 4–12% in spring and 1–9% in summer) (see Table S3, Supplementary Information). Because the crop production is sensitive to the solar radiation during the growing seaons^9^, the solar reduction (3-month prior to the harvest-time) is used for estimating the effect on the net crop yields. The difference in crop harvest time is considered (see Table S2, Supplementary Information). For example, in the southern part of the NCP region (e.g., Henan province), the wheat harvest-time is mainly in June; therefore the solar reductions in May, April, and March are used for the net yield estimation. In contrast, in the northern part of the NCP region (e.g., Hebei province), the wheat harvest-time is mainly in August; the solar reductions in July, June, and May are used for the net yield estimate. For rice, the main harvest-time is in July in the YRD, CEC, and SCB regions; the solar reductions in June, May, and April are used for the net yield estimate.

**Figure 3:**
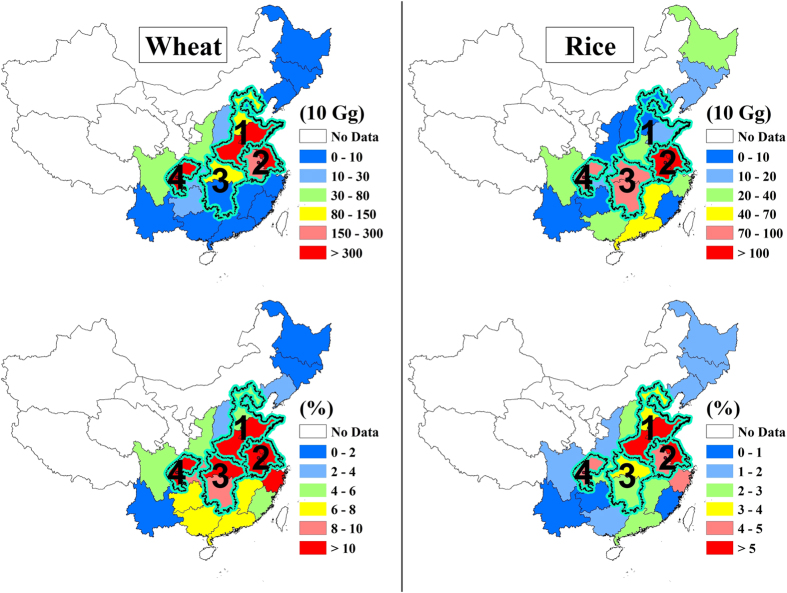
The estimates of the reductions of wheat (left panels) and rice (right panels) productions in China. The upper panels show the net yield reduction (10 Gg = 10^10^ g). The lower panels show the reduction of the net yield in percent. The map was generated by the IDL software version IDL 8.1 (Exelis, USA), http://www.exelisvis.com/.

Figure 3 shows the estimated reductions of wheat and rice production in China due to reduction in solar radiation, using the relationship between solar radiation and crop yields in China (see Table S4, Supplementary Information). Note that the relationship is derived from studies in Nanjing in central China^9^,^10^,^11^,^12^,^13^,^14^. Our results therefore should not be considered as a definitive answer because of the lack of crop-radiation data from region-specific field studies. However, our results show that the heavy aerosol pollution has significant impacts on the crop production in China. The wheat productions are estimated to be reduced by 10% in the NCP region, 13% in the YRD region, 11% in the CEC region, 14% in the SCB region, and 11% in the four regions on average. Because about 75% of wheat production is located in these four regions, the heavy aerosol pollution in these regions can lead to 8% (or 2.0 × 10^7^ tones) reduction in total wheat production in China. For the effect on rice production, it is estimated to be reduced by 6% in the NCP region, 6% in the YRD region, 3% in the CEC region, and 4% in the SCB region. On average, the rice reduction is 4% in the four regions, which accounts for 2% (or 5.9 × 10^6^ tones) reduction of total rice production in China because about 45% of rice production is located in these four regions.

However, there are large uncertainties, which need to be considered in this estimate. This estimate didn’t take into account the effect of diffuse solar radiation on the crop production. According to the study by Mercado *et al*.^15^, diffuse solar radiation has important impacts on total photo-synthetically active radiation (PAR). The fraction of diffuse solar radiation tends to increase photosynthesis compared to the direct solar radiation. However, there are large uncertainties in estimating the balance between the reduction of total PAR and the increase of PAR due to the diffuse fraction, which lead to uncertainties in estimating crop production. In this study, a sensitivity study was conducted using different diffuse solar radiation cases (low, moderate, and high cases). The results suggested that the diffuse solar radiation significantly decreased the estimated crop reductions. For example, under high diffuse radiation case, the estimated reductions were 1% and 4.5% for rice and wheat, respectively, which were much lower than the original estimate. The uncertainty of thick cloud was also estimated. Considering the fraction of thick cloud cover, the estimated reductions of rice and wheat were 0.9% and 3.4%, respectively (see Supplementary Information for the uncertainty estimate).

In general, our results suggest that the severe aerosol pollution can lead to significant reduction in wheat and rice production in China, which may offset the increase (~10–20%) from hybrid technology^22^. Mitigating regional aerosol pollution in China could therefore have the benefit of significantly increasing the crop yields in the nation. This has significant benefit for global food security, given a rapidly rising food demand in China in the coming decades.

## Methods

The satellite measurements of aerosol column distributions provided by the MODIS data were used in this study. The MODIS product monitors the ambient aerosol optical depth (AOD) globally. The monthly averaged AOD data with 0.1 × 0.1 degree horizontal resolution during April 2010 to April 2012 were used for this study. Because of the relatively high-resolution data, it has been under large influences of uncertainty factors, such as cloud covers, bright surface albedos, etc. As a result, the horizontal distributions of AOD exit a large number of miss points, producing a non-smooth spatial distributions. In order to have smoothed distributions, we make a further average of the data to reduce the horizontal resolution to 0.5 degree in longitude and latitude.

The effect of aerosol on solar radiative forcing was estimated using a state-of-the-art radiation transfer model (TUV). The TUV model is developed at National Center for Atmospheric Research^20^,^21^, and has been widely used by the scientific community. The detailed description of the model is also available from http://www.acd.ucar.edu/TUV. In this study, the model calculated the surface spectral irradiance for the wavelength range between 300 and 750 nm. The TUV model requires the following aerosol properties to study the aerosol effects on the surface UV irradiance, including (1) the horizontal distributions of AOD, (2) the normalized vertical profile of AOD, (3) the aerosol single albedo. A cloud-free condition is assumed for the calculation, because the AOD values are calculated based on cloud-free conditions. A surface albedo of 0.2 was assumed for all calculation. The monthly mean AOD horizontal distributions were used for the calculations. The normalized AOD vertical profile was calculated based on the measurement of aerosol vertical distribution, which was well mixed inside the PBL (Planetary Boundary Layer) height, and rapidly decreased outside of the PBL height^20^,^21^. The aerosol single albedo was assumed to be 0.80. The value is consistent to the composition measurements in Beijing, China^23^, in which a large amount of aerosol particles are scattering particles, such as sulfate, nitrate, ammonium, and organic carbon aerosols.

## Additional Information

**How to cite this article**: Tie, X. *et al*. Effect of heavy haze and aerosol pollution on rice and wheat productions in China. *Sci. Rep.* **6**, 29612; doi: 10.1038/srep29612 (2016).

## Supplementary Material

Supplementary Information
