# Supplementary material for: Effect of heavy haze and aerosol pollution on rice and wheat productions in China
Source: Sci Rep. 2016 Jul 8;6:29612. doi: 10.1038/srep29612 (PMC4937395; doi:10.1038/srep29612)
Supplement: Supplementary Information [file srep29612-s1.pdf]

## Supplementary Information for

# Effect of heavy haze and aerosol pollution on rice and wheat productions in China

Xuexi Tie[1,2], Ru-Jin Huang[1,3,4,*], Wenting Dai[1,*], Junji Cao[1,5*,], Xin Long[1], Xiaoli Su[1], Shuyu Zhao[1], Qiyuan Wang[1], Guohui Li[1]

[1]Key Laboratory of Aerosol Chemistry & Physics, SKLLQG, Institute of Earth Environment, Chinese Academy of Sciences, Xi'an, 710061, China

[2]CAS Center for Excellence in Urban Atmospheric Environment, Xiamen, 361021, China.

[3]Centre for Atmospheric and Marine Sciences, Xiamen Huaxia University, Xiamen 361024, China

[4]Laboratory of Atmospheric Chemistry, Paul Scherrer Institute (PSI), 5232 Villigen, Switzerland

[5]Institute of Global Environmental Change, Xi'an Jiaotong University, Xi'an, 710049, China

## 1. Information of the crop production in China

Table S1 and Figure S1 show the detailed crop production in China in 2012 (ref. 1).

**Table S1.** The rice and wheat production in China and the 4 regions studied.

| Production | Rice | | Wheat | |
|---|---|---|---|---|
| | (10 Gg) | (%) | (10 Gg) | (%) |
| China | 30057.0 | 100 | 24270.4 | 100 |
| 1-NCP | 845.6 | 2.8 | 11312.9 | 46.6 |
| 2-YRD | 4479.5 | 14.9 | 4495.4 | 18.5 |
| 3-CEC | 6102.5 | 20.3 | 1054.0 | 4.3 |
| 4-SCB | 2007.9 | 6.7 | 1259.3 | 5.2 |
| **Four regions** | **13435.4** | **44.7** | **18121.6** | **74.7** |

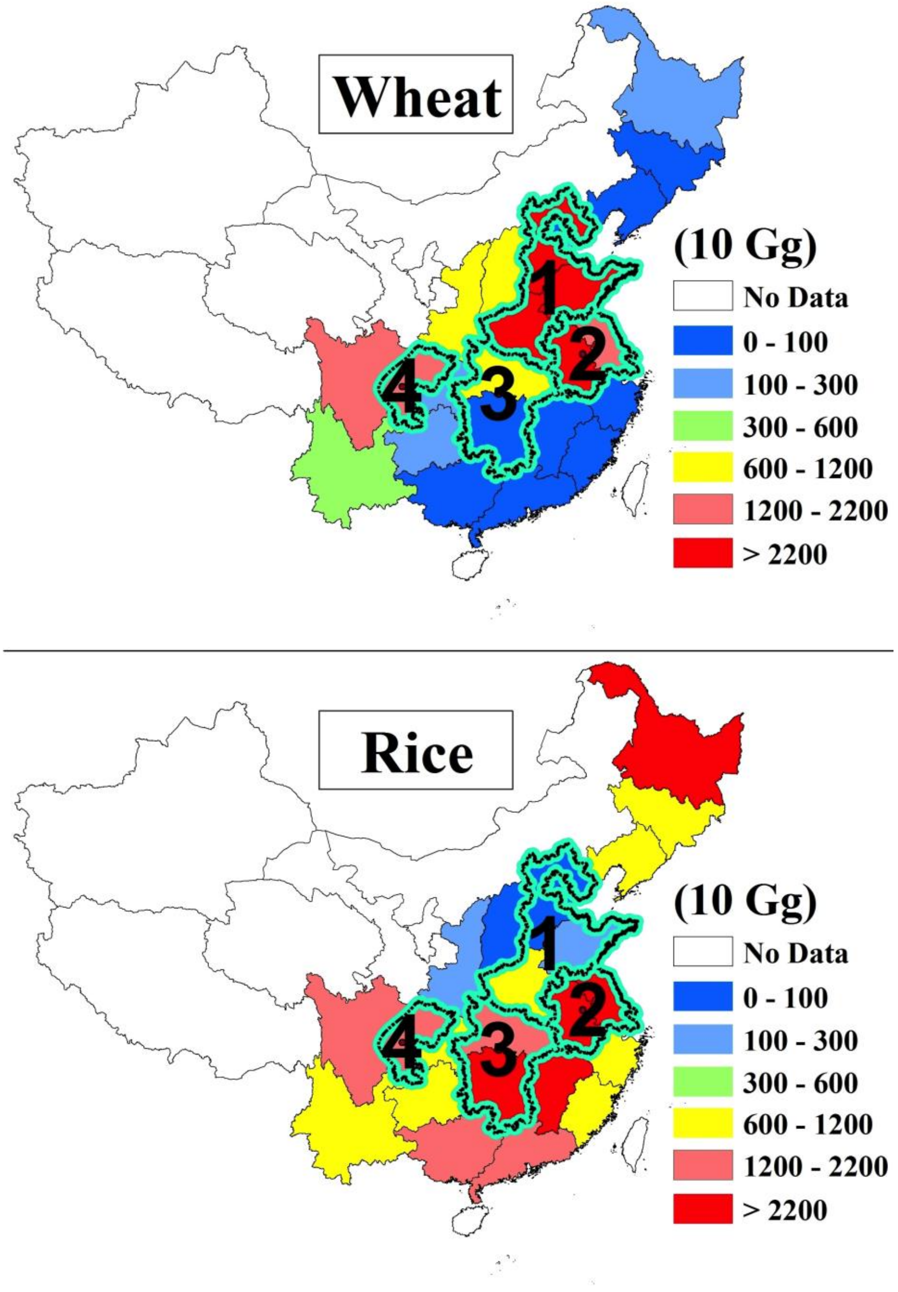


**Figure S1.** The distributions of rice and wheat production areas in China. The outlines with numbers represent the high AOD regions shown in Fig. 1. (1-NCP; 2-YRD; 3-CEC; 4-SCB). It shows that regions 1, 2, and 4 are the important wheat production areas, while regions 2, 3, and 4 are the important rice production areas. The map was generated by the IDL software version IDL 8.1 (Exelis, USA), http://www.exelisvis.com/.

**Table S2.** The rice and wheat production in the 4 studied regions.

| Region | Harvest-time | | Production (10 Gg) | | Delta (%) | | Delta (10 Gg) | |
|---|---|---|---|---|---|---|---|---|
| | Rice | Wheat | Rice | Wheat | Rice | Wheat | Rice | Wheat |
| Hebei | Oct | Aug | 83.0 | 2396.1 | 3.4 | 4.4 | 2.8 | 104.5 |
| Henan | Jul | Jun | 638.0 | 5323.3 | 5.8 | 10.2 | 37.0 | 543.9 |
| Shandong | Jun | May | 124.5 | 3593.5 | 8.1 | 13.8 | 10.1 | 497.5 |
| **1-NCP** | | | **845.6** | **11312.9** | **5.9** | **10.1** | **50.0** | **1146.0** |
| Jiangsu | Jul | May | 2248.6 | 2112.4 | 7.6 | 17.2 | 171.4 | 363.1 |
| Anhui | Jul | Jun | 2230.8 | 2383.0 | 4.9 | 10.0 | 109.6 | 239.4 |
| **2-YRD** | | | **4479.5** | **4495.4** | **6.3** | **13.4** | **281.0** | **602.5** |
| Hubei | Jul | May | 2036.2 | 1013.6 | 3.5 | 11.3 | 71.9 | 114.9 |
| Hunan | Jul | May | 4066.3 | 40.4 | 2.4 | 9.0 | 97.5 | 3.6 |
| **3-CEC** | | | **6102.5** | **1054.0** | **2.8** | **11.2** | **169.4** | **118.5** |
| **4-SCB** | **Jul** | **May** | **2007.9** | **1259.3** | **4.4** | **13.7** | **87.5** | **172.5** |

**Table S3** The statistical results of seasonal solar radiance reduction (%) in each region.

| Region | Winter | Spring | Summer | Autumn |
|---|---|---|---|---|
| **Minimum solar radiance reduction (%)** | | | | |
| **1-NCP** | 0.0 | 0.5 | 0.1 | 2.0 |
| **2-YRD** | 8.8 | 3.1 | 0.1 | 3.9 |
| **3-CEC** | 5.1 | 1.3 | 0.0 | 1.6 |
| **4-SCB** | 5.8 | 1.9 | 0.1 | 2.0 |
| **Mean solar radiance reduction (%)** | | | | |
| **1-NCP** | 16.4 | 8.3 | 6.5 | 11.6 |
| **2-YRD** | 20.3 | 10.5 | 6.2 | 13.3 |
| **3-CEC** | 15.4 | 6.5 | 2.4 | 7.8 |
| **4-SCB** | 22.0 | 8.4 | 4.4 | 9.3 |
| **Maximum solar radiance reduction (%)** | | | | |
| **1-NCP** | 49.0 | 22.9 | 17.7 | 32.0 |
| **2-YRD** | 35.1 | 18.3 | 14.4 | 20.7 |
| **3-CEC** | 27.8 | 14.9 | 8.9 | 15.8 |
| **4-SCB** | 34.8 | 16.9 | 10.6 | 17.9 |
| **First quartile (Q1) of solar radiance reduction (%)** | | | | |
| **1-NCP** | 11.0 | 5.1 | 4.5 | 7.2 |
| **2-YRD** | 17.9 | 9.7 | 4.1 | 12.6 |
| **3-CEC** | 11.3 | 3.6 | 0.9 | 5.1 |
| **4-SCB** | 16.6 | 6.6 | 2.4 | 6.3 |
| **Third quartile (Q3) of solar radiance reduction (%)** | | | | |
| **1-NCP** | 21.4 | 11.2 | 8.7 | 15.5 |
| **2-YRD** | 23.5 | 12.3 | 7.8 | 15.1 |
| **3-CEC** | 19.4 | 8.8 | 3.7 | 10.0 |
| **4-SCB** | 29.2 | 10.5 | 6.3 | 12.4 |

## 2. Relationship between crop production and solar radiation

**Table S4.** Reduction of the rice and wheat net yields corresponding to the changes of solar radiation at the surface. The data are extracted from Chameides et al. (ref. 2), based on several in-situ field measurements[3-7].

| The percent of Observed irradiance (UV) change (%) | Crop yield change (%) | |
|---|---|---|
| | Rice | Wheat |
| 0 | 0 | 0 |
| -5 | -3.75 | -5.25 |
| -10 | -7.5 | -10.5 |
| -15 | -11.25 | -15.75 |
| -20 | -15.0 | -21.0 |
| -25 | -18.75 | -26.25 |
| -30 | -22.5 | -31.5 |

### 2.1 Uncertainties of diffuse radiation on the estimate of crop production.

The aerosol particles have two important effects on the surface solar radiation. First, aerosol particles reduce the total photo-synthetically active radiation (PAR) on the surface. Second, aerosol particles partially change direct solar radiation to diffuse solar radiation. According to the previous studies[8,9], the diffuse solar radiation has important impacts on photosynthesis. The fraction of diffuse solar radiation tends to increase photosynthesis compared to the direct solar radiation. However, there are large uncertainties in estimating the balance between the reduction of total PAR and the increase of PAR due to the diffuse fraction, which lead to uncertainties in estimating crop production. In this study, uncertainties of the solar effects with different diffuse solar radiation for crop production were estimated. The ratios (R= DI/DU) between direct and diffuse solar radiation were measured in different regions of the world, ranging from 2.5 to 7.0 (ref. 9). Where DI and DU represent direct and diffuse solar radiation, respectively. In this estimate, R=2.5, 5.0 and 7.0 represent high,

moderate, and low diffuse solar radiation. In Table S4, the relationship between solar radiation and crop production was measured in the US crop field, which was consistent to the low diffuse case (R=7). For the moderate (R=5) and high (R=2.5) diffuse cases, we first calculate the fraction of diffuse radiation, using the different ratios of R. Then the higher yield of crop production from diffuse radiation than direct radiation was estimated according to the study of Mercado et al. (Fig. 1a of ref. 8). Their study suggested that diffuse radiation had higher production rate of gross primary productivity (GPP) than direct radiation, which was applied in the estimate for the crop production. The higher potions of the diffuse radiation for the crop production were calculated with the moderate (R=5) and high (R=2.5) diffuse cases than the base case (low diffuse radiation; R=7). Table S5 shows the aerosol effects on the crop production with the different diffuse cases. This uncertainty study shows that the diffuse radiation could have important impacts for the estimate of crop reduction due to aerosol particles. For example, in the most rice production region (CEC), the estimated reduction of rice varied from 2.8% (low diffuse case) to 0.7% (high diffuse case), indicating that the diffuse solar radiation tended to increase crop production. Overall, the estimated rice reductions were 2.0, 1.4, and 1.0% under the low, moderate, and high diffuse radiation cases, respectively. The estimated wheat reductions were 8.4, 5.8, and 4.5% under the low, moderate, and high diffuse radiation cases, respectively.

**Table S5.** Estimate of the reduction (%) of rice and wheat, with different diffuse solar radiation in different regions.

| Regions | Production (10 Gg) | | Reduction (%) | |
|---|---|---|---|---|
| | Rice | Wheat | Rice | Wheat |
| | | | R=7 (L) | |
| 1-NCP | 845.6 | 11312.9 | 5.9% | 10.1% |
| 2-YRD | 4479.5 | 4495.4 | 6.3% | 13.4% |
| 3-CEC | 6102.5 | 1054.0 | 2.8% | 11.2% |
| 4-SCB | 2007.9 | 1259.3 | 4.4% | 13.7% |
| **Region** | **13435.4** | **18121.6** | **4.4%** | **11.3%** |
| **China** | **30057.0** | **24270.4** | **2.0%** | **8.4%** |
| | | | R=5 (M) | |
| 1-NCP | 845.6 | 11312.9 | 4.8% | 6.6% |
| 2-YRD | 4479.5 | 4495.4 | 5.1% | 10.0% |
| 3-CEC | 6102.5 | 1054.0 | 1.6% | 7.7% |
| 4-SCB | 2007.9 | 1259.3 | 3.2% | 10.3% |
| **Region** | **13435.4** | **18121.6** | **3.2%** | **7.8%** |
| **China** | **30057.0** | **24270.4** | **1.4%** | **5.8%** |
| | | | R=2.5 (H) | |
| 1-NCP | 845.6 | 11312.9 | 2.7% | 5.4% |
| 2-YRD | 4479.5 | 4495.4 | 4.3% | 7.5% |
| 3-CEC | 6102.5 | 1054.0 | 0.7% | 5.2% |
| 4-SCB | 2007.9 | 1259.3 | 2.3% | 7.9% |
| **Region** | **13435.4** | **18121.6** | **2.3%** | **6.1%** |
| **China** | **30057.0** | **24270.4** | **1.0%** | **4.5%** |

## 2.2 Uncertainties of cloud cover for the estimate of crop production.

Our based calculation assumes that the calculated solar reduction due to aerosol was under clear-sky condition. However, there were thick cloud covers in fraction of time. Under thick cloud cover conditions, the calculated changes of solar radiation due to aerosol particles were overestimated. The uncertainties due to cloud cover should be estimated. Under thick cloud cover condition, the solar reduction was estimated by $\Delta Y = \Delta X \times (1.0 - f(\text{cloud}))$. Where $\Delta X$ and $\Delta Y$ represent the reduction of solar radiation under clear sky and under cloudy conditions, respectively, and $f$(cloud) represents the fraction of thick cloud cover. Figure S2 shows the measured probability of thick cloud cover in different seasons. In this study, the fraction of thick cloud cover was

estimated based on the level-3 MODIS data, with daily 1 x 1 degree grid average values of atmospheric thick cloud fraction. The thick cloud was defined when cloud top was greater than 700 mb. Figure S2 shows that there was a large seasonal variation of cloud fraction. Because the harvest seasons of rice and wheat were different, the fraction of cloud cover was also different as shown in Table S6. The fraction of thick cloud cover ranged from 5.3 to 7.0% and 9.1 to 18.0% for the estimate rice and wheat reductions, respectively. As a result, in considering with the cloud fraction, the estimated rice reduction decreased to 1.8, 1.3, and 0.9% for the low, moderate, and high diffuse radiation, respectively. The estimated wheat reduction decreased to 7.4, 5.1, and 3.4% for the low, moderate, and high diffuse radiation, respectively.

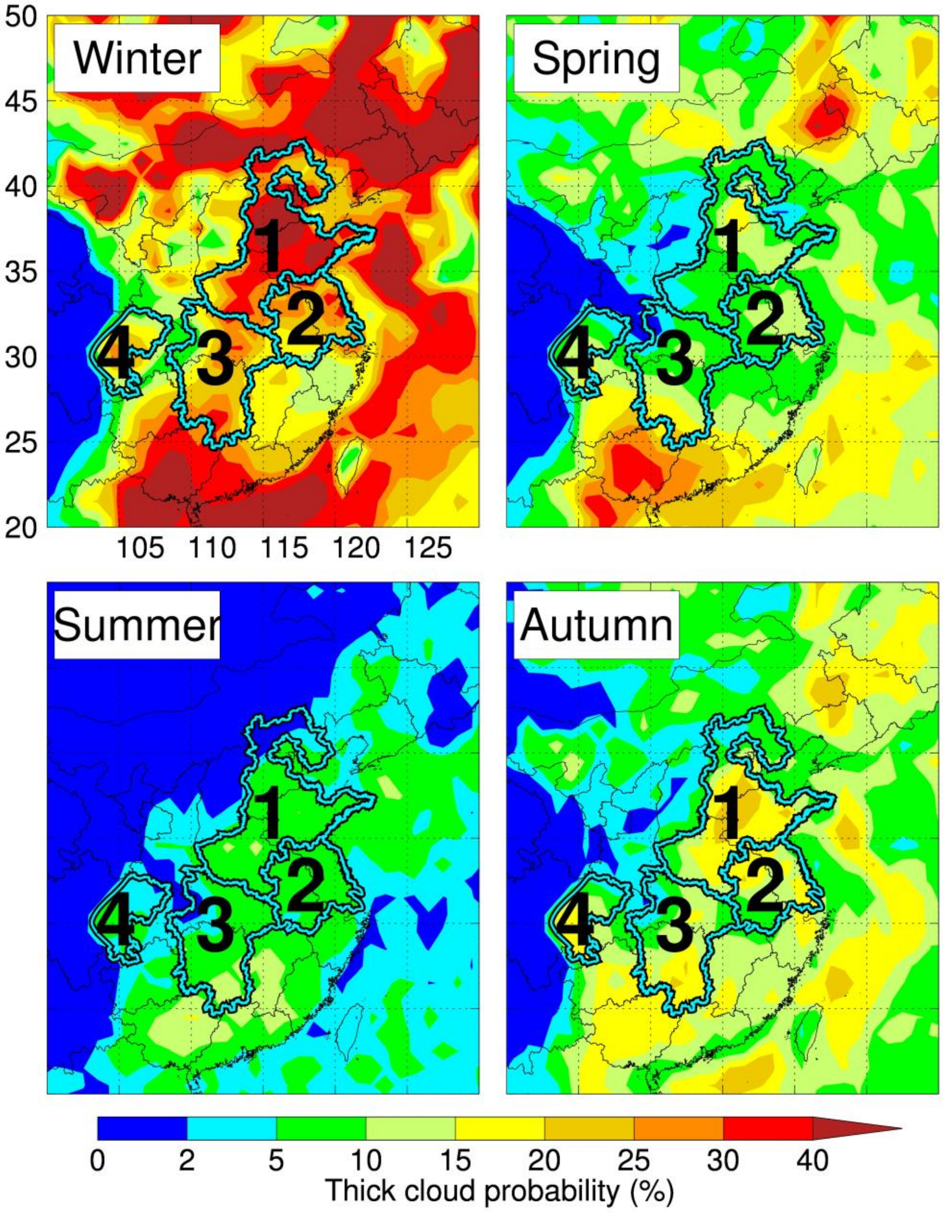


**Figure S2.** Estimates of thick cloud cover probability in different regions. The estimate is based on level-3 MODIS data, with daily 1 x 1 degree grid average values of atmospheric thick cloud fraction.

**Table S6.** Estimate of the reduction (%) of rice and wheat, with different diffuse solar radiation in different regions, including the cloud condition.

| Regions | **Reduction** (%) **Without Cloud** | | **Cloud Cover** (%) | | **Reduction** (%) **With Cloud** | |
|---|---|---|---|---|---|---|
| | Rice | Wheat | Rice | Wheat | Rice | Wheat |
| | **R=7 (L)** | | | | **R=7 (L)** | |
| 1-NCP | 5.9% | 10.1% | 5.3% | 9.1% | 5.6% | 9.2% |
| 2-YRD | 6.3% | 13.4% | 7.0% | 13.8% | 5.8% | 11.5% |
| 3-CEC | 2.8% | 11.2% | 5.6% | 18.0% | 2.6% | 9.2% |
| 4-SCB | 4.4% | 13.7% | 5.8% | 11.0% | 4.1% | 12.2% |
| **Region** | **4.4%** | **11.3%** | | | **4.1%** | **9.9%** |
| **China** | **2.0%** | **8.4%** | | | **1.8%** | **7.4%** |
| | **R=5 (M)** | | | | **R=5 (M)** | |
| 1-NCP | 4.8% | 6.6% | 5.3% | 9.1% | 4.5% | 6.0% |
| 2-YRD | 5.1% | 10.0% | 7.0% | 13.8% | 4.8% | 8.6% |
| 3-CEC | 1.6% | 7.7% | 5.6% | 18.0% | 1.5% | 6.3% |
| 4-SCB | 3.2% | 10.3% | 5.8% | 11.0% | 3.0% | 9.2% |
| **Region** | **3.2%** | **7.8%** | | | **3.0%** | **6.9%** |
| **China** | **1.4%** | **5.8%** | | | **1.3%** | **5.1%** |
| | **R=2.5(H)** | | | | **R=2.5(H)** | |
| 1-NCP | 2.7% | 5.4% | 5.3% | 9.1% | 2.6% | 4.9% |
| 2-YRD | 4.3% | 7.5% | 7.0% | 13.8% | 4.0% | 6.5% |
| 3-CEC | 0.7% | 5.2% | 5.6% | 18.0% | 0.7% | 4.3% |
| 4-SCB | 2.3% | 7.9% | 5.8% | 11.0% | 2.2% | 7.0% |
| **Region** | **2.3%** | **6.1%** | | | **2.2%** | **4.5%** |
| **China** | **1.0%** | **4.5%** | | | **0.9%** | **3.4%** |

## References


1. The annual statistics book of China, 2012, shown in http://www.stats.gov.cn/tjsj/ndsj/2012/indexch.htm

2. Chameides, W.L., H. Yu, S.C. Liu, M. Bergin, X. Zhou, L. Mearns, G. Wang, C.S. Kiang, R.D. Saylar, C. Lou, Y. Huang, A. Steiner, and F. Giorgi, Case study of the effects of atmospheric aerosols and regional haze on agriculture: An opportunity to enhance crop yields in China through emission controls? *P. Natl. Acad. Sci.*, 96, 13626-13633, 1999.

3. Stansel, J. & Huke, R., in *Impacts of Climatic Change on The Biosphere, Part 2: Climatic Effects*, ed. Bartholic, J. R. (U.S. Department of Transportation, Washington,

DC), DOT-TST-75-55, pp. 4.90-4.130, 1975.

4. Yoshida, S. & Paro, F. T., in *Climate and Rice* (International Rice Research Institute, Los Banos, Phillippines), pp. 187-210, 1976.

5. Robertson, G. W., *WMO Technical Note 144* (World Meteorological Organization, Geneva), pp. 1-40, 1975.

6. Islam, M. & Morrison, J. I. L., *Field Crop. Res.* **30,** 13-28, 1992.

7. Lomas, J., *WMO Technical Note* (World Meteorological Organization, Geneva) pp. 1–30, 1976.

8. Mercado, L. M., Bellouin, N., Sitch, S., Boucher, O., Huntingford, C,, Wild, M. & Cox, P., Impact of changes in diffuse radiation on the global land carbon sink, *Nature*, **458,** 1014-1018, 2009.

9. Liepert, B. & Tegen, I. Multidecadal solar radiation trends in the United States and Germany and direct tropospheric aerosol forcing. *J. Geophys. Res.* **107,** doi:10.1029/2001JD000760, 2002.
